# Development of a Stromal Microenvironment Experimental Model Containing Proto-Myofibroblast Like Cells and Analysis of Its Crosstalk with Melanoma Cells: A New Tool to Potentiate and Stabilize Tumor Suppressor Phenotype of Dermal Myofibroblasts

**DOI:** 10.3390/cells8111435

**Published:** 2019-11-14

**Authors:** Angelica Avagliano, Maria Rosaria Ruocco, Rosarita Nasso, Federica Aliotta, Gennaro Sanità, Antonino Iaccarino, Claudio Bellevicine, Gaetano Calì, Giuseppe Fiume, Stefania Masone, Mariorosario Masullo, Stefania Montagnani, Alessandro Arcucci

**Affiliations:** 1 Department of Public Health, University of Naples Federico II, 80131 Naples, Italy; 2 Department of Molecular Medicine and Medical Biotechnology, University of Naples Federico II, 80131 Naples, Italy; 3 Department of Movement Sciences and Wellness, University of Naples ‘Parthenope’, 80133 Naples, Italy; 4 IEOS Istituto di Endocrinologia e Oncologia Sperimentale ‘G. Salvatore’, National Council of Research, 80131 Naples, Italy; 5 Department of Experimental and Clinical Medicine, University of Catanzaro ‘Magna Graecia’, Viale Europa, 88100 Catanzaro, Italy; 6 Department of Clinical Medicine and Surgery, University of Naples Federico II, 80133 Naples, Italy

**Keywords:** melanoma, stromal microenvironment, conditioned medium, proto-myofibroblasts

## Abstract

Melanoma is one of the most aggressive solid tumors and includes a stromal microenvironment that regulates cancer growth and progression. The components of stromal microenvironment such as fibroblasts, fibroblast aggregates and cancer-associated fibroblasts (CAFs) can differently influence the melanoma growth during its distinct stages. In this work, we have developed and studied a stromal microenvironment model, represented by fibroblasts, proto-myofibroblasts, myofibroblasts and aggregates of inactivated myofibroblasts, such as spheroids. In particular, we have generated proto-myofibroblasts from primary cutaneous myofibroblasts. The phenotype of proto-myofibroblasts is characterized by a dramatic reduction of α-smooth muscle actin (α-SMA) and cyclooxygenase-2 (COX-2) protein levels, as well as an enhancement of cell viability and migratory capability compared with myofibroblasts. Furthermore, proto-myofibroblasts display the mesenchymal marker vimentin and less developed stress fibers, with respect to myofibroblasts. The analysis of crosstalk between the stromal microenvironment and A375 or A2058 melanoma cells has shown that the conditioned medium of proto-myofibroblasts is cytotoxic, mainly for A2058 cells, and dramatically reduces the migratory capability of both cell lines compared with the melanoma-control conditioned medium. An array analysis of proto-myofibroblast and melanoma cell-conditioned media suggests that lower levels of some cytokines and growth factors in the conditioned medium of proto-myofibroblasts could be associated with their anti-tumor activity. Conversely, the conditioned media of melanoma cells do not influence the cell viability, outgrowth, and migration of proto-myofibroblasts from spheroids. Interestingly, the conditioned medium of proto-myofibroblasts does not alter the cell viability of both BJ-5ta fibroblast cells and myofibroblasts. Hence, proto-myofibroblasts could be useful in the study of new therapeutic strategies targeting melanoma.

## 1. Introduction

Melanoma is one of the most metastasizing and drug resistant solid tumors and is characterized by an impressive plasticity influenced by the interaction between cancer cells and the stromal microenvironment that regulates melanoma growth, progression and therapeutic response [1]. The cancer stromal microenvironment can be formed by normal fibroblasts, fibroblast aggregates, and cancer-associated fibroblasts (CAFs) [1,2,3,4,5,6].

Fibroblasts, in vivo, are present in normal connective tissue and express, as well as myofibroblasts, the mesenchymal marker vimentin [7,8]. Fibroblasts belong to a heterogeneous population of mesenchymal cells characterized by an exceptional phenotypic plasticity and the capability to secrete large amounts of soluble factors, extracellular matrix (ECM) components, and extracellular vesicles. These cells can regulate the physiological turnover of the ECM, but they can also participate in wound healing and senescence [6]. In particular, during the initial phase of wound healing, the migration of fibroblast into the ECM that occurs before myofibroblast differentiation represents a fundamental process of wound contraction [9]. In fact, when the wound begins to close, migrating cells develop tractional forces that sustain the closure process. During this process, mechanical stress sustains the differentiation of fibroblasts into proto-myofibroblasts [7]. Proto-myofibroblasts are characterized by a cytoskeleton with stress-fibers lacking α-smooth muscle actin (α-SMA) and a proliferative and migratory program [7,10]. During wound healing, in many fibro-contractive diseases and even in developing and specialized normal tissues, mechanical stress and specific factors can induce the expression of α-SMA and the differentiation of proto-myofibroblasts into myofibroblasts. However, the presence of proto-myofibroblasts in some adult tissues suggests that they can persist and function as an independent cell type without differentiating into myofibroblasts [7]. Myofibroblasts represent activated fibroblasts, are characterized by a cytoskeleton with extensively developed stress fibers and α-SMA expression, and are devoted to contraction and ECM synthesis during wound remodeling and contraction [7,10,11]. Moreover, it is known that myofibroblasts can acquire an immunoregulatory phenotype and produce large amounts of cyclooxygenase-2 (COX-2) and pro-inflammatory cytokines [12,13]. After wound closure, myofibroblasts undergo apoptotic cell death or revert to quiescent fibroblasts. Conversely, during chronic inflammation, myofibroblasts continue to produce the ECM leading to fibrotic tissue that is also present in desmoplastic solid tumors [14].

It is known that fibroblasts can modulate melanoma development differently. In particular, normal dermal fibroblasts can block melanoma formation during its early stage, whereas senescent fibroblasts sustain melanoma growth [2,15,16,17]. Moreover, at the early stages of melanoma, fibroblasts form aggregates in the dermis, and a paracrine interaction with melanoma cells leads to CAF differentiation and the formation of a dermal tumor niche [4]. In particular, the interaction between melanoma cells and fibroblast aggregates induces fibroblast reprogramming that is linked to an increase of migratory capability [4]. Therefore, in solid tumors such as melanoma, infiltrated and surrounding fibroblasts are stimulated to differentiate into constitutively-activated CAFs that share similarities with myofibroblasts [6]. CAFs represent the most prominent non-cancer cell type within the reactive stroma of many solid tumors, coevolving with the disease and modifying the biochemical and physical structure of the tumor microenvironment [6]. It is noteworthy that, compared with normal wound healing process, tissues of solid tumors can be considered wounds that never heal [18]. In this scenario, both α-SMA and COX-2 proteins represent important markers for CAF differentiation [6,19]. Furthermore, in melanoma tissue, CAFs form a network that provides the building blocks for macromolecule biosynthesis and ATP production, both of which are necessary for the proliferation and growth of cancer cells [6,20], and they sustain through paracrine signals the progression of metastatic melanoma [16,21,22].

Though the influence of CAFs on melanoma growth and outcome is known [23,24,25], the impact of normal fibroblasts on solid tumors, such as melanoma, is still little understood. For this reason, we focused our in vitro study on the bidirectional interaction between melanoma cells and the stromal microenvironment, represented by fibroblasts in various differentiation stages and fibroblasts aggregates.

We have previously shown that the in vitro formation of aggregates such as spheroids from primary cutaneous myofibroblasts leads to a dramatic decrease of α-SMA and COX-2 levels in fibroblasts within spheroids [26]. Both the decrease of α-SMA and COX-2 in protein extracts of spheroids and the cytostatic effect exerted by the conditioned medium of spheroids on both normal and cancer cell lines have indicated that myofibroblasts undergo a deactivation process within spheroids. Additionally, α-SMA levels and a confocal microscopy analysis of cytoskeleton of fibroblasts from spheroids reverted to adhesion growth have suggested that their phenotype resembles that of proto-myofibroblasts [26].

Therefore, in this work, we developed an in vitro experimental system, resembling a part of the stromal microenvironment, to study its interaction with melanoma cells. In particular, we generated proto-myofibroblast-like cells from primary cutaneous myofibroblasts, and we have provided evidence for myofibroblast de-activation in vitro, supporting the concept that myofibroblasts are not terminally-differentiated cells [27,28]. Moreover, our study indicates that myofibroblast dedifferentiation may be pivotal to potentiate the fibroblasts anti-tumor activity. In fact, we show a cytotoxic and anti-migratory effect exerted by the proto-myofibroblast-conditioned medium on both A375 and A2058 melanoma cells.

An array analysis of proto-myofibroblast and melanoma cell-conditioned media has indicated that the decrease of some cytokines and growth factor secretion in a proto-myofibroblast-conditioned medium could be associated with the capability of proto-myofibroblasts to hinder the viability and migratory capability of melanoma cells.

This experimental system could represent a new tool to elucidate the processes that regulate the crosstalk between melanoma cells and the stromal microenvironment. Additionally, proto-myofibroblasts could represent a cell type that is useful to study new therapeutic strategies targeting melanoma.

## 2. Materials and Methods

### 2.1. Cutaneous Tissues

Normal skin specimens were obtained from donors (n = 11 females; mean age 46.7 ± 5.6) who had undergone neck surgery for benign pathology, such as multinodular goiter without thyroiditis. Patients with metabolic and connective tissue diseases were excluded. The investigation conformed to the principles outlined in the Declaration of Helsinki [29], and informed consent was obtained from all the patients. The study reported in the manuscript received the approval from the Ethic Committee of the University of Naples Federico II (Comitato Etico Università Federico II). The assigned protocol number of the study is 228/18.

### 2.2. Cell Cultures, Spheroids Generation and Preparation of Conditioned Medium

Cutaneous myofibroblasts and spheroids were obtained and cultured as previously described [26]. Reverted fibs were obtained from spheroids that were transferred to plastic culture plates, expanded, and then maintained in culture as bidimensional (2D) monolayers for about 30 days to allow for the achievement of a stable phenotype. All the experiments were performed only with cells from the early passage (<9).

A375 and A2058 human melanoma cells and the human foreskin fibroblast cell line BJ-5ta cells were kindly provided by CEINGE (Naples, Italy). Cells were grown in DMEM supplemented with 10% FBS, 2 mM l-glutamine, 100 IU/mL penicillin G and 100 μg/mL streptomycin (standard culture medium) in a humidified incubator at 37 °C under a 5% CO_2_ atmosphere. All cells were split, seeded every 3 days, and used during their exponential phase of growth. Cell treatments were usually carried out 24 h after plating. The cells were observed with a CKX41 model Olympus phase contrast microscope (Olympus Corporation, Tokyo, Japan); the images were acquired by a camera connected to the microscope by means of Cell-A software. Melanoma cell lines and all the fibroblastic cell types (1 × 10^4^ cells/well) were seeded in 96-well plate and cultured in a standard medium cell culture. After 48 h, 50 µL of a fresh standard cell culture medium were added in each well. Then, conditioned media were collected after 72 h and stored at −20 or −80 °C until use. To exclude that the observed effects on melanoma cell viability and migration were due to the amounts of serum in conditioned media, we refreshed proto-myofibroblast and melanoma cell-conditioned media by adding the same amount (10%) of fresh serum before performing ATP and wound healing assays.

For the spheroid-conditioned medium preparation, myofibroblasts were grown as spheroids, and the medium was collected after 72 h of 3D culture. Alternatively, for the spheroid outgrowth experiments, 1 × 10^6^ A375 and A2058 cells were seeded in 10 cm cell culture dishes and incubated in a cell culture standard medium for 24 h. Therefore, the conditioned medium was collected at 24 h.

All chemicals were of analytical grade and were purchased from Sigma-Aldrich (St. Louis, MO, USA).

### 2.3. Total Cell Lysates and Western Blotting Analysis

A total cell lysate analysis and a western blotting analysis of monolayer cells and spheroids were performed as previously described [26]. The membranes were incubated overnight at 4 °C with the following primary antibodies: COX-2 (Abcam, Cambridge, UK and Elabscience Biotechnology Inc. Houston, TX, USA) α-SMA (Abcam, Cambridge, UK and Elabscience Biotechnology Inc. Houston, TX, USA), vimentin (Cell Signal Technology Inc., Danvers, MA, USA) and GAPDH (Cell Signal Technology) and then with horseradish peroxidase-linked specific secondary antibodies (Santa Cruz Biotechnology, Santa Cruz, CA, USA) at room temperature for 1 h. The membranes were analyzed by an enhanced chemiluminescence reaction, using Super Signal West Femto Maximum Sensitivity Substrate kit (Thermo Scientific, Waltham, MA, USA) according to the manufacturer’s instructions. The signals were visualized by autoradiography. The images were acquired by Epson Perfection 2480 Photo (Epson, Suwa, Japan), and a densitometric analysis was performed using the Image J software, version 1.48i (Wayne Rasband National Institutes of Health, Bethesda, MD, USA).

### 2.4. Immunohistochemistry

Each spheroid was fixed in 10% neutral buffered formalin and embedded in paraffin (Bio-Optica Milano SpA, Milan, Italy) and then sliced into serial 4 μm thick sections and placed on poly-l-lysine-coated glass slides (Menzel-Glaser, Brunswick, Germany). The slides were deparaffinized, rehydrated and immersed in 10 mM citric acid with a pH of 6 (Sigma-Aldrich) in a microwave oven (VWR International PBI Srl, Milan, Italy) for three cycles of 5 min at 650 Watt in order to exclude epitope masking owing to fixation. Spheroids sections were hematoxylin and eosin stained for necrosis analysis. Other sections were immunostained with primary antibodies against vimentin (Ventana Medical Systems, Tucson, AZ, USA) detected by the Ultra View Universal DAB Detection Kit (Ventana Medical Systems), according to the manufacturer’s protocol. Therefore, these microscopic slides were digitized into high-resolution files through a NanoZoomer-2.0RS digital scanner (Hamamatsu, Tokyo, Japan).

### 2.5. Wound Healing and Cell Invasion Assays

A wound healing assay was performed by seeding 2.5 × 10^5^ cells into 35 mm tissue culture plates and growing them to confluence. Then, the confluent monolayer of cells was carefully scratched with a sterilized pipette tip, and, after wounding, the plates were rinsed twice with 1 × PBS to remove cell debris. Cells were incubated with the conditioned media of all types of fibroblast and melanoma cells. Photographs of the same fields were taken at 0 and 48 h with a camera connected to a CKX41 model Olympus phase contrast microscope, and the images were acquired using the Cell-A software. A quantitative analysis of the open wound was performed by measuring the gap area at different time points using the Image J software, version 1.48i (National Institute of Health, Bethesda, MD, USA). The ratio between the relative open surface area at indicated time points and at baseline was calculated.

A cell invasion assay was carried out in 24-well matrigel invasion chambers (Corning BioCoat Matrigel Invasion Chamber, Bedford, MA, USA). In brief, A375 and A2058 cells were seeded in invasion chambers (upper chambers, 2 × 10^4^ cells per 24-well invasion chamber) in a serum-free medium. The lower chambers were filled with BJ-5ta, proto-myofibroblasts, myofibroblasts and spheroid-conditioned media; A375- or A2058-conditioned media were used as controls. Following culturing at 37 °C and 5% CO_2_ for 24 h, the cells that did not migrate through the matrigel membrane were subsequently removed with a cotton swab, and the cells at the bottom of the membrane were stained with Diff-Quik Stain Set (Medion Diagnostics, Miami, FL, USA). Then, randomly chosen fields were photographed (×10), and the number of invaded cells was calculated as a percentage of invasion. The images were taken in bright field with a digital camera (Leica DC200; Leica Microsystems, Milan, Italy) connected to the microscope (Leica DMLB; Leica Microsystems).

All chemicals were of analytical grade and were purchased from Sigma-Aldrich.

### 2.6. ATP Assay

To evaluate the effect of BJ-5ta cells, proto-myofibroblasts, myofibroblasts, spheroids and melanoma cell-conditioned media on cell viability, 1 × 10^4^ fibroblasts or melanoma cells/well were seeded in 96-well plates and let to grow for 24 h. Then, the culture medium was removed and replaced with 100 μL of the conditioned medium. After 48 h of incubation, an ATP assay was performed by a CellTiter-Glo^®^ Luminescent Cell Viability Assay kit (Promega, Madison, WI, USA), according to the manufacturer’s protocol.

### 2.7. Immunofluorescence and Confocal Microscopy

A morphological analysis of proto-myofibroblasts and myofibroblasts was performed as previously described [26]. In particular, the cells were plated on glass coverslips, fixed with 4% paraformaldehyde, permeabilized with 0.1% Triton X-100, blocked in donkey serum (Millipore, Billerica, MA, USA), and diluted to 1:10 in 1× PBS for 30 min at room temperature. Glass coverslips were incubated with a mouse monoclonal anti-vimentin primary antibody (Sigma-Aldrich), diluted 1:50 for 1 h at 37 °C, washed three times with 1× PBS, and then subsequently incubated with an FITC donkey, anti-mouse secondary antibody (Jackson ImmunoResearch, Suffolk, UK) diluted to 1:50 and phalloidin-TRITC (Sigma-Aldrich) diluted to 1:100 for 1 h at 37 °C. The cell nuclei were labelled with DAPI (Vector Laboratories, Inc, Burlingame, CA, USA). Glass coverslip mounting was done in Vectashield (Vector Laboratories). The immunofluorescence and fluorescence analyses of both proto-myofibroblasts and myofibroblasts were carried out on an inverted and motorized microscope (Axio Observer Z.1) equipped with a 63×/1.4 Plan-Apochromat objective. The attached laser-scanning unit (LSM 700 4× pigtailed laser 405-488-555-639; Zeiss, Jena, Germany) enabled confocal imaging. For excitation, 405, 488, and 555 nm lasers were used. Fluorescence emission was revealed by a main dichroic beam splitter and a variable secondary dichroic beam splitter. Triple staining fluorescence images were acquired separately using the ZEN Black 2012 software in the red, green and blue channels at a resolution of 512 × 512 pixels, with the confocal pinhole set to one Airy unit and then saved in the TIFF format.

All other chemicals were of analytical grade and were purchased from Sigma-Aldrich.

### 2.8. Cytofluorimetric Analysis of Cell Death

To evaluate cell death, 2 × 10^4^ melanoma cells/well were seeded into 96-well/plates; at the end of incubation with conditioned media, cell suspensions were centrifuged, and pellets were resuspended in a hypotonic lysis solution containing 50 mg/mL of propidium iodide (PI). After incubation at 4 °C for 30 min, cells were analyzed by flow cytometry to evaluate the number of nuclei with a DNA content lower than the diploid [30].

All chemicals were of analytical grade and were purchased from Sigma-Aldrich.

### 2.9. Spheroid Migration Assay

The area covered by fibroblast cells migrating out from the spheroids and spreading on a plastic surface was used as an index of cell migration [31]. Each fibroblast spheroid was transferred to an individual well of a 24-well plate and incubated with a standard cell culture medium or melanoma cell-conditioned media. The images of the three different types of spheroids were captured at 0 and 24 h and acquired with a camera connected to a CKX41 model Olympus phase contrast microscope using the Cell-A software. The migration area was determined by calculating the difference between the final area (24 h) and the initial area (0 h) using the Image J analysis program.

### 2.10. Cytokines and Growth Factors Profiling of Conditioned Media

Cytokines, chemokines and growth factors secreted by melanoma cells and proto-myofibroblasts were detected in conditioned media (CM) by using a RayBio cytokine array-C3 (RayBiotech, Inc. Peachtree Corners, GA, USA). Conditioned media were collected and subjected to a 42 biological factors profile according to the manufacturer’s instructions. The signal intensity of each spot, which represents the secreted chemokines, cytokines, and growth factors, was evaluated by subtracting the background and normalized to positive controls using the Image J software.

### 2.11. Statistical Analysis

Numerical data were reported in Kaleida Grafh 4.0 and analyzed by a Student’s t-test; a one-way ANOVA, with Bonferroni corrections, was used for multiple comparisons.

## 3. Results

### 3.1. Characterization of Fibroblast Population in the Stromal Microenvironment

**Figure 1:**
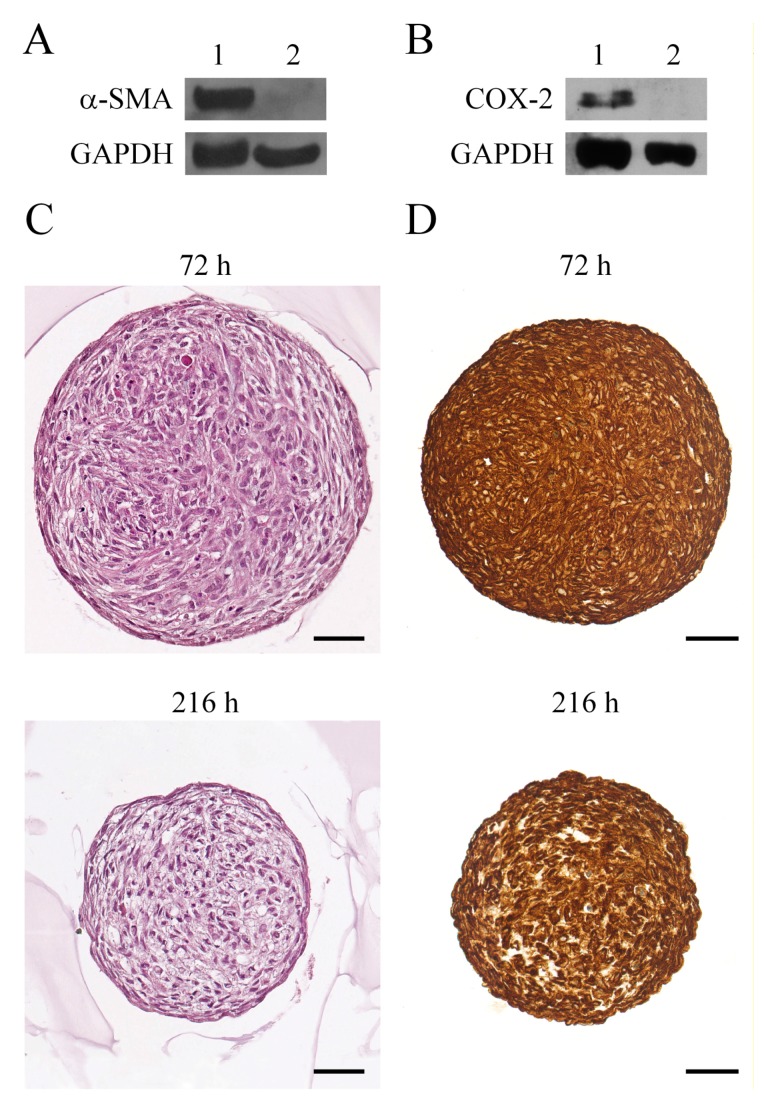
Evaluation of activation markers in Bj-5ta cells and an analysis of myofibroblasts spheroids collected at 72 and 216 h. Western blotting analysis of α-smooth muscle actin (α-SMA) (**A**) and cyclooxygenase-2 (COX-2) (**B**) in protein extracts of cutaneous myofibroblasts (1) and BJ-5ta cells (2). GAPDH was used as the loading control. A representative image of three independent experiments is shown. (**C**) Haematoxylin and eosin staining and (**D**) vimentin immunohistochemical analysis of paraffin-embedded sections of spheroids collected at 72 and 216 h. The slides were digitized into high-resolution files. Scale bar 50 μm. Magnification ×40. All images are representative of three independent experiments.

We in vitro developed a part of the stromal microenvironment comprised of inactivated fibroblasts, proto-myofibroblasts, myofibroblasts, and fibroblast aggregates. As inactivated fibroblasts, we chose the BJ-5ta cell line [32] that does not embody the key features of activated fibroblasts but expresses vimentin, a typical marker of mesenchymal cells such as fibroblasts [33,34]. In particular, BJ-5ta cells, an immortalized skin fibroblasts cell line, do not express α-SMA and COX-2 (Figure 1A,B), markers of myofibroblasts [26].

The absence of both α-SMA and COX-2 in the protein extracts of BJ-5ta cells indicated that these cells represent inactivated fibroblasts.

Then, after 216 h of 3D culture (reverted fibs), we deepened the phenotype study of cells from spheroids transferred to cell culture dishes and reverted to adhesion growth to compare the levels of activation, inflammation and mesenchymal markers with respect to myofibroblasts grown as monolayer and spheroids. To this aim, we generated spheroids from human primary cutaneous myofibroblasts [26], and we collected the spheroids after 72 and 216 h of 3D culture. In our previous work, we demonstrated that spheroids collected at 72 and 216 h show an impressive and comparable reduction of α-SMA levels with respect to the myofibroblast monolayer [26]. Therefore, in the present study, we compared the presence of necrosis areas that could be associated with the inflammation process [35] and vimentin markers in spheroids collected at 72 and 216 h. To this aim, we performed the hematoxylin and eosin staining and vimentin immunostaining of spheroids (Figure 1C,D). This analysis did not detect any area of necrosis in spheroids collected at both 72 and 216 h (Figure 1C), thus excluding the presence of the inflammation process. Additionally, an immunohistochemical analysis showed the presence of vimentin in spheroids collected at different time points (Figure 1D). Moreover, it is noteworthy that the decrease of spheroid volume over time is due, as previously described, to a compaction process [26]. In the complex, these data indicated that spheroids collected at both 72 and 216 h are comparable regarding the presence of activation, inflammation and mesenchymal markers, and these spheroids could equally be used as fibroblast aggregates in our experimental system.

**Figure 2:**
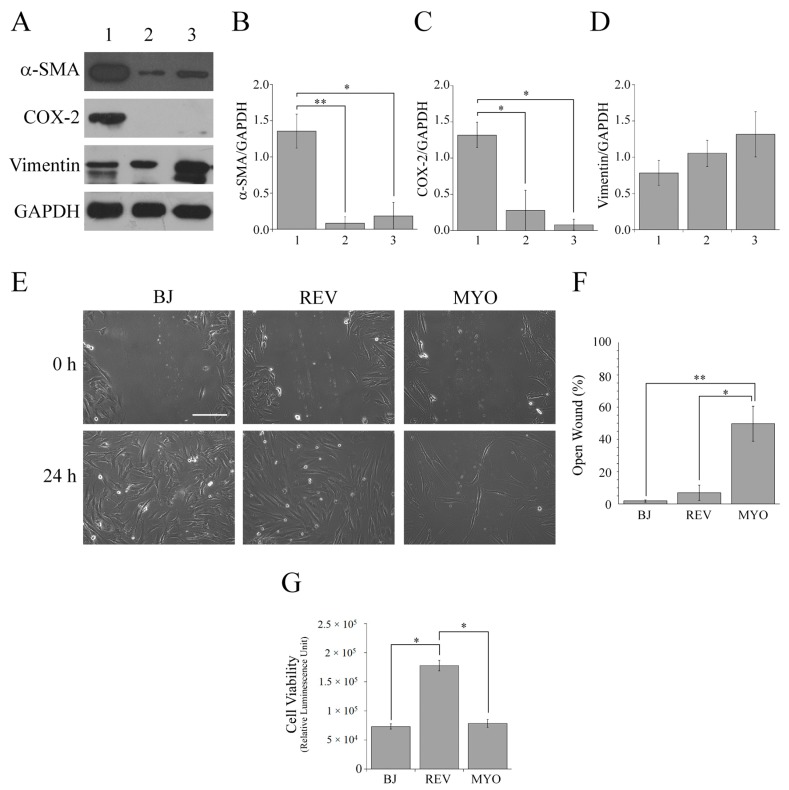
The characterization of the fibroblast population in the stromal microenvironment. (**A**) Western blotting analysis of α-SMA, COX-2 and vimentin in protein extracts of myofibroblasts (1), spheroids collected at 72 h (2) and reverted fibs (3). GAPDH was used as the loading control. A representative image of three independent experiments is shown. (**B**–**D**) Densitometric analyses of α-SMA, COX-2 and vimentin protein levels. Data are reported as means of three independent experiments ± S.E. * *p* < 0.05, ** *p* < 0.01. (**E**) The evaluation of migratory capability of BJ-5ta (BJ), reverted fibs (REV) and myofibroblast (MYO) cells by a wound healing assay. (**F**) The quantification of the wound healing assay. Wound widths were measured at 0 and 24 h after wounding. Data are expressed as percentage of the fold-decrease of the open wound area compared with the control (0 h), set as 100%, and they are reported as a mean of three independent experiments ± S.E. * *p* < 0.05, ** *p* < 0.01. (**G**) The evaluation, by an ATP assay, of the cell viability of BJ-5ta (BJ), reverted fibs (REV) and myofibroblasts (MYO) cells incubated for 48 h with a standard culture medium. Data are means of at least three independent experiments ± S.E. * *p* < 0.0001.

Hence, by western blotting the protein levels of α-SMA, COX-2 and vimentin in extracts of cutaneous myofibroblasts to analyze the spheroids collected at 72 h and which were reverted fibs (Figure 2).

This analysis detected a significant decrease of both α-SMA and COX-2 protein levels in reverted fibs and spheroids compared with myofibroblasts, but it did not show any difference between reverted fibs and spheroid cells. On the other hand, significant differences of vimentin levels were not detected (Figure 2A–D). Moreover, it is important to note the remarkable standard error of the densitometric analysis of reverted fibs α-SMA and COX-2 levels (Figure 2B) due to the presence of specimens that do not express the proteins. Hence, the significant differences in α-SMA and COX-2 levels indicate that myofibroblasts, spheroid cells and reverted fibs represent distinct states of fibroblast differentiation.

It is known that α-SMA expression in fibroblasts leads to a decrease of motility [36] and that fibroblasts, during their differentiation stages, display different migratory capabilities [7]. Therefore, we evaluated the migratory capability of BJ-5ta, reverted fibs and myofibroblast cells by wound healing assays (Figure 2E,F). This analysis detected a greater wound healing capability of both BJ-5ta cells and reverted fibs compared with myofibroblasts. In particular, at 24 h after wounding, the quantitative analysis (Figure 2F) indicated that in both BJ-5ta and reverted fibs cultures, the scratch area was almost closed. Conversely, at the same time point, in the myofibroblast culture, the percentage of open surface area was still about of 50%. The significant greater migratory capability of both BJ-5ta cells and reverted fibs compared with myofibroblasts can be explained by very low levels of α-SMA in both the BJ-5ta cells and reverted fibs compared with myofibroblasts. Additionally, the observed differences in migratory capabilities also sustain the distinct differentiation stages of the three fibroblasts cell types [7].

It is known that an ATP cell viability assay can be used for measuring cell proliferation rate [37]. An ATP cell viability assay performed on BJ-5ta, reverted fibs and myofibroblast cells incubated with a cell culture standard medium showed that the cell viability of reverted fibs is significantly greater than that of both BJ-5ta and myofibroblast cells (Figure 2G). These data indicate that reverted fibs have a greater proliferation rate compared with both BJ-5ta and myofibroblast cells.

**Figure 3:**
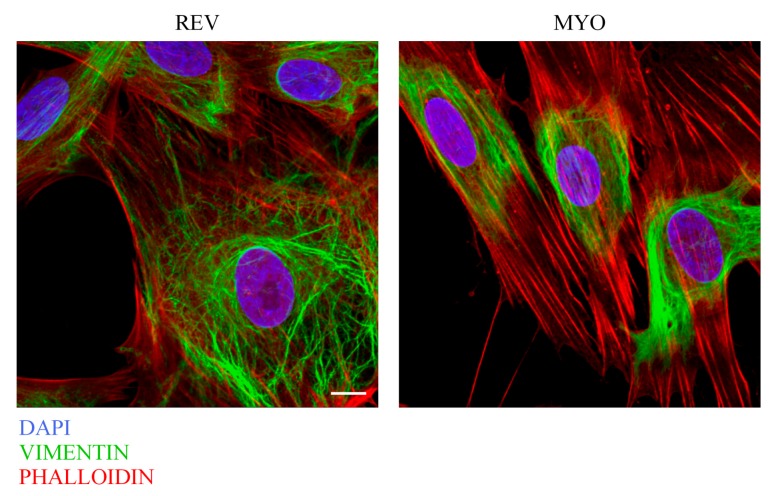
A cytoskeleton analysis of reverted fibs and myofibroblast cells. A confocal fluorescence analysis of vimentin immunostaining (green channel) and phalloidin staining (red channel) of reverted fibs (REV) and myofibroblast (MYO) cells. DAPI (blue channel) was used to locate the nuclei. Images correspond to the 3D reconstruction of Z-planes acquired from the top to the bottom of the cell. Scale bar 10 µm. The images are representative of three independent experiments.

Therefore, we compared the cytoskeleton organization of reverted fibs and myofibroblast cells by confocal fluorescence and immunofluorescence analyses (Figure 3).

In both fibroblast cell-types, the presence of vimentin intermediate filaments and stress fibers were evident, but the reverted fibs displayed a cytoskeleton organization resembling that of proto-myofibroblasts, with fewer and less developed stress fibers compared with the myofibroblast monolayer.

In the complex, the absence of α-SMA and COX-2 expression, the low cell viability, and the greater motility of BJ-5ta cells compared with myofibroblasts confirmed that BJ-5ta cells represent inactivated fibroblasts. Furthermore, the dramatic decrease of α-SMA and COX-2 levels, the greater migratory capability, the higher cell viability, and the different stress fibers pattern of reverted fibs cells compared with myofibroblasts allowed us to identify reverted fibs as proto-myofibroblast-like cells [7,10]. Hence, hereafter, we refer to reverted fibs as proto-myofibroblast cells.

### 3.2. Influence of the Stromal Microenvironment on Viability, Migration and Invasion of Melanoma Cells

It is known that the interaction between melanoma cells and the microenvironment sustains metabolic reprogramming, melanoma growth, and cancer progression [1,4,15,16,21,22]. In particular, during melanoma development, skin fibroblasts undergo a differentiation into CAFs that is induced by an interaction with malignant melanocytes [1]. Conversely, the influence of normal stromal fibroblasts on solid tumors such as melanoma is largely unexplored and poorly understood.

**Figure 4:**
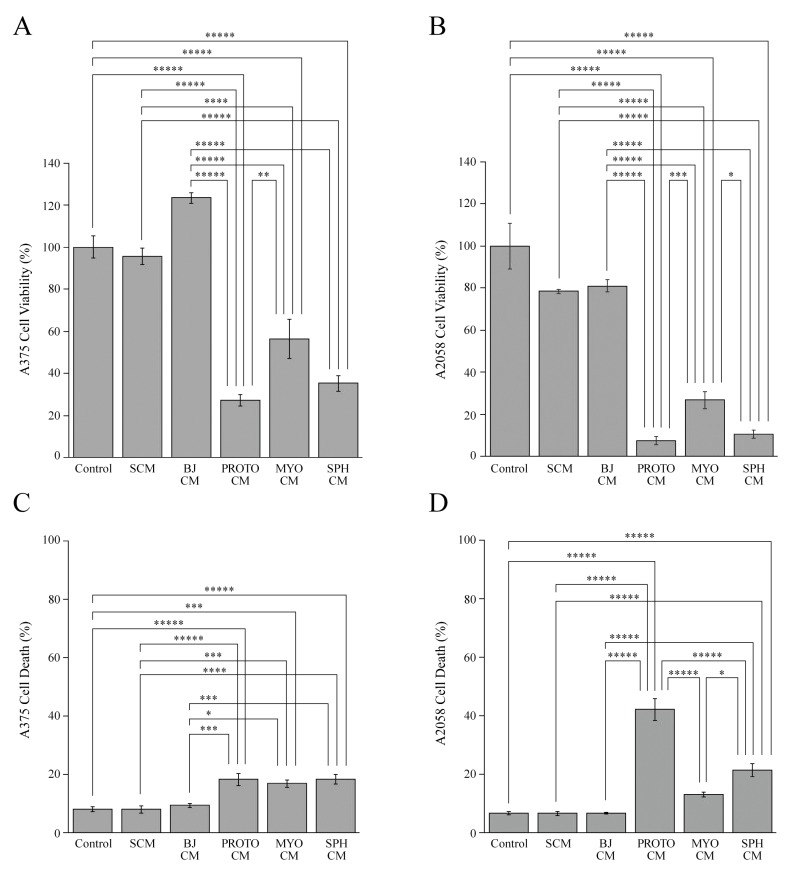
The influence of the stromal microenvironment on the viability of melanoma cells. The cell viability and cytotoxic effect of a standard culture medium (SCM), the conditioned media of BJ-5ta (BJ CM), proto-myofibroblast (PROTO CM), myofibroblast (MYO CM) and spheroid (SPH CM) cells on A375 (**A**,**C**) and A2058 melanoma cells (**B**,**D**). The controls are represented by A375 or A2058 cells incubated with their own conditioned medium. A375 and A2058 cells were grown for 48 h with standard culture medium and conditioned media, and then cell viability and the cytotoxic effect were evaluated by an ATP assay and a cytofluorimetric analysis of the number of nuclei with a subdiploid DNA content, respectively. Data are means of at least three independent experiments ± S.E. * *p* < 0.05, ** *p* < 0.01, *** *p* < 0.005, **** *p* < 0.0005, ***** *p* < 0.0001.

Therefore, we first evaluated the effect of the conditioned media of BJ-5ta, proto-myofibroblasts, myofibroblast and spheroid cells on the viability, migration and invasion of A375 and A2058 melanoma cells in order to study the bidirectional interaction between these cell components of stroma and melanoma cells. The controls are represented by A375 and A2058 cells treated with their own conditioned media. To evaluate the viability of melanoma cells, we performed ATP cell viability assays on melanoma cells treated with the stromal-conditioned media. In particular, A375 cells treated with the conditioned media of proto-myofibroblasts, myofibroblasts and spheroids showed a significant viability reduction of about 80%, 50% and 70%, respectively, compared with the control (Figure 4A).

On the other hand, the conditioned medium of BJ-5ta cells did not significantly affect the viability of melanoma cells. It is noteworthy that A375 cells incubated with a standard culture medium, containing 10% fresh serum, did not display any difference of cell viability compared with cells incubated with their own conditioned medium. This experimental evidence indicates that diverse amounts of serum contained in melanoma cell-conditioned media and the standard culture medium influenced A375 cell viability to the same extent.

To further evaluate if different amounts of serum present in conditioned media could affect the cell viability of melanoma cells, we analyzed the cell viability of A375 cells incubated with their own conditioned medium, with or without addition of 10% fresh serum, with proto-myofibroblast-conditioned medium, and with or without addition of 10% fresh serum (Figure S1A). There were no differences in the viability of A375 cells treated with proto-myofibroblast-conditioned medium and the same conditioned medium supplemented with fresh serum. In the same way, no differences in cell viability were detected between A375 cells treated with their own conditioned medium (control) and the cells incubated with the same conditioned medium supplemented with fresh serum. On the contrary, a significant decrease in cell viability was observed in A375 cells treated with proto-myofibroblast-conditioned media, with or without addition of fresh serum, compared with melanoma cells exposed to their own conditioned media supplemented or not with fresh serum (Figure S1A). These results are consistent with our previous data, showing an anti-proliferative effect exerted by proto-myofibroblasts on A375 cells.

To investigate if this reduction of cell viability was associated with cell death, we evaluated the effect of conditioned media on the number of nuclei with a sub-diploid content through a cytofluorimetric analysis (Figure 4C). Moreover, we also verified the influence of serum amounts in conditioned media on cell death. To this aim we have compared cell death percentage of melanoma cells treated with a standard culture medium with respect to melanoma cells incubated with their own conditioned medium.

This analysis showed a significant increase of cell death in A375 cells incubated with the conditioned media of proto-myofibroblasts, myofibroblasts and spheroids, compared with control and melanoma cells treated with a standard culture medium or BJ-5ta cell-conditioned medium. It is important to note that A375 cells treated with their own conditioned medium or with a standard culture medium did not show differences in cell death. Therefore, differences in serum amounts did not affect melanoma cell death. Moreover, the trend of cell viability and cell death was comparable. The conditioned media of proto-myofibroblasts, myofibroblasts and spheroids also dramatically and significantly reduced the viability of A2058 cells compared with the control (Figure 4B). In particular, proto-myofibroblast-, myofibroblast- and spheroid-conditioned media reduced A2058 cell viability at rates of about 95%, 80% and 90%, respectively. Conversely, A2058 cells exposed to their own conditioned medium, standard culture medium or BJ-5ta-conditioned medium did not show significant difference in cell viability. It is noteworthy that the A2058 cell-conditioned medium and standard culture medium affected cell viability to a similar extent: this experimental proof strongly indicates that the different serum amounts present in conditioned media did not influence melanoma cell viability. This hypothesis was further confirmed by evaluating the cell viability of A2058 cells incubated with the conditioned media of cancer cells or proto-myofibroblasts, with or without the addition of 10% fresh serum (Figure S1B). A significant decrease in cell viability was detected in A2058 cells treated with proto-myofibroblast-conditioned media, supplemented or not with fresh serum, compared with melanoma cells incubated with their own conditioned media, with or without the addition of fresh serum (Figure S1B). Conversely, no difference was observed in cell viability between A2058 cells treated with the proto-myofibroblast-conditioned medium and the same cells incubated with the proto-myofibroblast-conditioned medium supplemented with 10% fresh serum. Therefore, this analysis demonstrated that 10% fresh serum added to the conditioned media did not modify melanoma cell viability and, in particular, did not influence the dramatic cytostatic capacity of the proto-myofibroblast-conditioned medium.

Moreover, the analysis of A2058 cell numbers with a sub-diploid DNA content detected an impressive and significant cell death increase of melanoma cells treated with proto-myofibroblast and spheroid-conditioned media compared with the control and melanoma cells treated with the BJ-5ta cell-conditioned medium (Figure 4D). It is noteworthy that melanoma cells incubated with the conditioned media of proto-myofibroblasts and spheroids displayed a 6-fold and 3-fold increase of cell death, respectively, compared with the control and A2058 treated with the standard culture medium or the BJ-5ta cell-conditioned medium. No differences in cell death were observed between A2058 cells treated with their own medium, a standard culture medium and the conditioned media of BJ-5ta cells and myofibroblasts. In particular, the A2058 cell-conditioned medium and standard culture medium affected melanoma cell death to the same extent.

**Figure 5:**
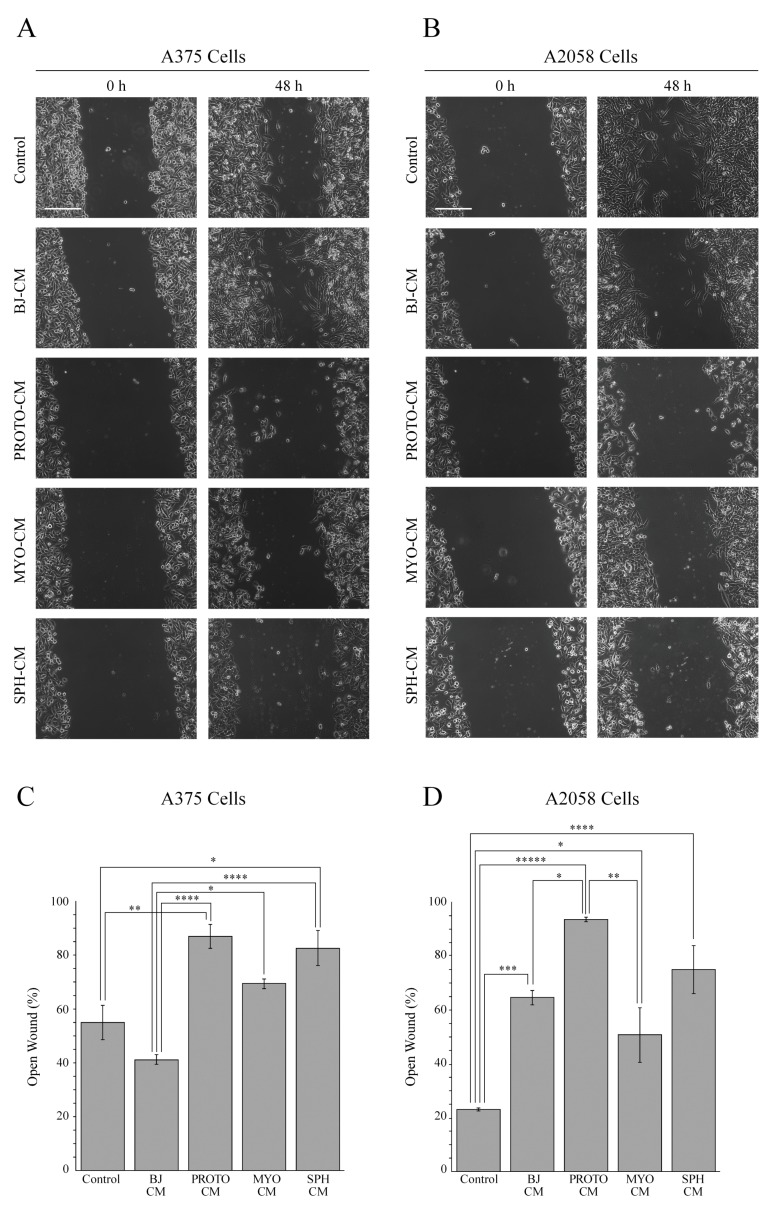
The influence of the stromal microenvironment on the migration of melanoma cells. The wound healing assay of A375 (**A**) and A2058 (**B**) cells, grown for 48 h with the conditioned media of BJ-5ta (BJ CM), proto-myofibroblast (PROTO CM), myofibroblast (MYO CM) and spheroid (SPH CM) cells. Controls are represented by A375 and A2058 cells incubated with their own conditioned medium. The representative images of three independent experiments show the same fields with scratching at 0 and 48 h after wounding. Scale bar 200 µm. Magnification ×10. Migratory capability quantification of A375 (**C**) and A2058 (**D**) melanoma cells. Wound widths were measured at 0 and 48 h after wounding. Data are expressed as percentage of fold-decrease of open wound area compared with control (0 h) set as 100%, and are reported as mean of three independent experiments ± S.E. * *p* < 0.05, ** *p* < 0.005, *** *p* < 0.001, **** *p* < 0.0005, ***** *p* < 0.0001.

Therefore, we evaluated the effect of stromal microenvironment-conditioned media on the migratory capability of both low metastatic A375 and high metastatic A2058 melanoma cells [38] through wound healing assays (Figure 5A–D).

This analysis showed a significant decrease of the migratory capability of A375 cells incubated with proto-myofibroblast- and spheroid-conditioned media compared with the control and BJ-5ta-conditioned medium-treated cells (Figure 5A,C). On the other hand, the conditioned medium of myofibroblasts did not significantly affect the migration of A375 cells compared with the control-treated cells. Of special note is the fact that the quantitative analysis showed that 48 h after wounding in A375 culture exposed to proto-myofibroblast- and spheroid-conditioned media, the scratch area was almost open, whereas in A375 cells that were cultured with control conditioned medium, the open surface area was about 55% (Figure 5C).

The migratory capability of A2058 cells treated with conditioned media of BJ-5ta, proto-myofibroblasts, myofibroblasts and spheroids was significantly decreased compared with that of melanoma cells treated with their own conditioned medium (Figure 5B,D). In particular, 48 h after wounding, the quantitative analysis (Figure 5D) showed a percentage of open surface area in A2058 cells containing their own conditioned medium of about 20%. Conversely, at the same time point, in the A2058 culture treated with the BJ-5ta, proto-myofibroblast-, myofibroblast- and spheroid-conditioned media, the percentage of open wound areas was about 60%, 90%, 50% and 75%, respectively. It is noteworthy that the conditioned medium of the proto-myofibroblasts at 48 h almost completely blocked the migration of A2058 cells, as also observed for A375 cells (Figure 5D,C).

Moreover, we analyzed the influence of different serum amounts of melanoma cell- and proto-myofibroblast-conditioned media on the migratory capability of both A375 and A2058 cells by a wound healing assay. To this aim, we evaluated the migratory capacity of melanoma cells incubated with aliquots of conditioned media with or without addition of 10% fresh serum (Figure S2). No differences in cell motility were detected between A375 cells treated with the proto-myofibroblast-conditioned medium and cells incubated with the same conditioned medium supplemented with 10% fresh serum. In the same way, no differences in wound healing capability were observed between A375 cells incubated with their own conditioned medium (control) and cells exposed to the same conditioned medium supplemented with 10% fresh serum. Conversely, the conditioned media of proto-myofibroblasts, supplemented or not with fresh serum, significantly decreased the migratory capability of A375 cells compared with the same cells treated with their own conditioned media with or without addition of fresh serum (Figure S2A,C). Therefore, the addition of 10% fresh serum to the proto-myofibroblast-conditioned medium did not modify the anti-migratory effect exerted by this fibroblast cell type on A375 cells. The same analysis performed on A2058 cells showed that the addition of 10% fresh serum in the conditioned media of proto-myofibroblasts and melanoma cells did not influence the migratory capability of melanoma cells (Figure S2B,D). Indeed, there were no differences in cell migration between A2058 cells treated with the proto-myofibroblast-conditioned medium and melanoma cells incubated with the same medium supplemented with fresh serum. Similarly, A2058 cells incubated with their conditioned medium (control) did not show a significant difference in migratory capability compared with cells treated with the A2058 cell-conditioned medium supplemented with fresh serum. Proto-myofibroblast-conditioned media, with or without addition of fresh serum, significantly decreased the wound healing capability of A2058 cells compared with melanoma cell-conditioned media, supplemented or not with fresh serum.

Therefore, different amounts of serum present in the conditioned media of melanoma cells and proto-myofibroblasts did not influence either the viability or the migratory capability of cancer cells.

Invasion assays performed on A375 and A2058 cells, incubated with the different stromal-conditioned media, did not show any difference in the invasiveness capability of melanoma cells (not shown).

### 3.3. Influence of Melanoma Cell Signals on the Stromal Microenvironment

During melanoma progression, skin fibroblasts undergo a differentiation into CAFs due to their interaction with malignant melanocytes [1]. The CAF phenotype is associated with a metabolic reprogramming and slower proliferation rate compared with normal fibroblasts [6]. The aggressiveness of solid tumors depends on the capability of cancer cells to recruit the surrounding stromal cells, such as CAFs, which then can be found at the cancer invasive front. CAFs can be present, together with circulating cancer cells, in the peripheral blood of patients with metastatic cancer [19,39]. CAFs, or CAF aggregates, could provide a provisional stroma and establish a metastatic niche that facilitates growth and metastasis formation by moving from primary tumor to the secondary site [40,41]. Hence, CAF migratory capability, associated with a specific migratory, adhesive and paracrine signaling apparatus, could be important for cancer metastatic progression [42,43].

In this scenario, the reprogramming of normal fibroblasts, induced by their interaction with cancer cells, could lead to CAF formation, melanoma progression and dissemination [4,44].

**Figure 6:**
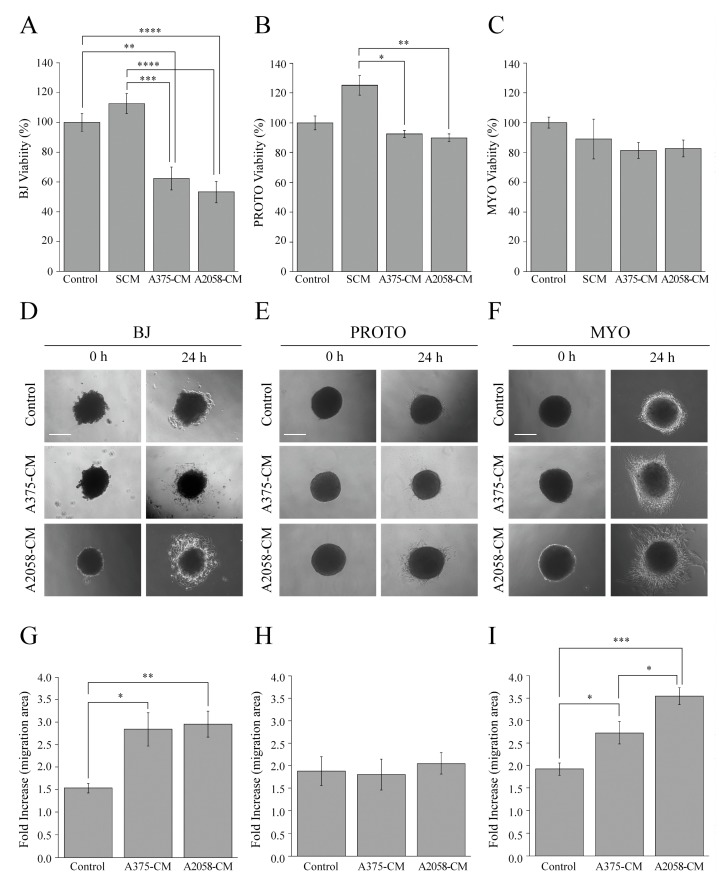
The effect of melanoma cell signals on stromal microenvironment. Influence of the standard culture medium (SCM), the conditioned media of A375 (A375 CM) and A2058 (A2058 CM) cells on viability of (**A**) BJ-5ta (BJ), (**B**) proto-myofibroblast (PROTO), and (**C**) myofibroblast (MYO) cells. Controls are represented by BJ-5ta, proto-myofibroblast or myofibroblast cells incubated with their own conditioned medium. The cells were grown for 48 h with a standard culture medium or conditioned media, and cell viability was evaluated by an ATP assay. Data are means of at least three independent experiments ± S.E. * *p* < 0.01, ** *p* < 0.005, *** *p* < 0.0005, **** *p* < 0.0001. The influence of conditioned media of A375 (A375 CM) and A2058 (A2058 CM) cells on cell outgrowth and migration from spheroids of (**D**) BJ-5ta (BJ), (**E**) proto-myofibroblast (PROTO), and (**F**) myofibroblast (MYO) cells. Controls are represented by spheroids exposed to a standard culture medium (Control). The quantification of the area covered by BJ-5ta (**G**), proto-myofibroblast (**H**) and myofibroblast (**I**) cells migrating out from the spheroids and spreading on a plastic surface. Data are means of at least three independent experiments ± S.E. * *p* < 0.05, ** *p* < 0.005, *** *p* < 0.0001.

Therefore, to analyze the influence of melanoma cell signals on fibroblast stromal microenvironment, we incubated BJ-5ta, proto-myofibroblast and myofibroblast cells with the conditioned medium of A375 or A2058 cells, and we evaluated metabolic activity and cell viability, as well as the outgrowth and migratory capability of the different fibroblasts cell types from spheroids. Moreover, we also treated the fibroblasts of the stromal microenvironment with a standard culture medium. Our results indicated that BJ-5ta cells treated with A375 and A2058 cell-conditioned media showed a decrease of metabolic activity and cell viability compared with the control (Figure 6A).

On the contrary, the conditioned media of melanoma cells did not exert any effect on the metabolic activity and cell viability of both proto-myofibroblasts and myofibroblasts (Figure 6B,C). Moreover, the different serum amounts present in the standard culture medium and in the conditioned media of all fibroblast cell types influenced the viability of stromal cells equally (Figure 6A–C).

It is known that fibroblast aggregates are formed in the dermis during melanoma development [4]. The interaction between melanoma cells and fibroblast aggregates leads to fibroblast reprogramming that is associated with increase of migratory capability, feature of CAFs [4]. Therefore, to evaluate if the paracrine interaction between melanoma cells and aggregates of stromal cells, such as spheroids, leads to an increase of fibroblast migration, feature of CAFs, BJ-5ta, proto-myofibroblast and myofibroblast cells, grown as spheroids for 216 h, were transferred on the standard culture dishes to allow for cell adhesion, outgrowth, and migration in the presence of the conditioned media of melanoma cells or a standard culture medium (Control) for 24 h. The conditioned media of melanoma cells did not influence the outgrowth and the migration of proto-myofibroblasts from the spheroids (Figure 6E,H), whereas a significant induction of the outgrowth and migration of both BJ-5ta (Figure 6D,G) and myofibroblast (Figure 6F,I) cells was observed. In particular, BJ-5ta spheroids treated for 24 h with the control cell culture medium and the conditioned media of A375 or A2058 cells, showed 1.5-, 2.8-, and 2.9-fold increases in their migration areas, respectively, compared with spheroids at 0 h (Figure 6G). Moreover, myofibroblast spheroids treated with the control medium or the A375 conditioned medium showed 1.9- and 2.6-fold increase in their migration areas, respectively. The highest increase in myofibroblast spheroids migration area, 3.6-fold, was observed after 24 h of incubation with the A2058 conditioned medium (Figure 6I). It is noteworthy that the interaction between proto-myofibroblasts and the conditioned media of melanoma cells did not affect cell viability, outgrowth and migration of proto-myofibroblasts.

### 3.4. Effect of Stromal Signals on Viability of Stromal Cells

**Figure 7:**
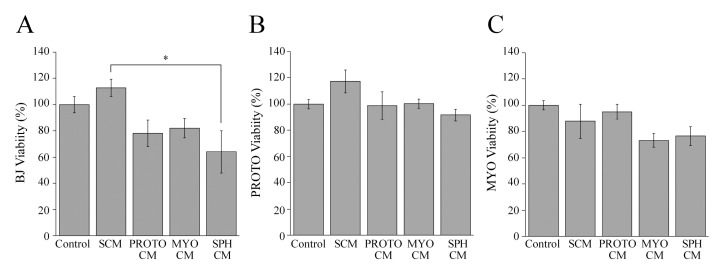
The influence of stromal microenvironment signals on fibroblast monolayer viability. The effect of the standard culture medium (SCM), the conditioned media of BJ-5ta (BJ CM), proto-myofibroblast (PROTO CM), myofibroblast (MYO CM), and spheroid (SPH CM) cells on the viability of (**A**) BJ-5ta (BJ), (**B**) proto-myofibroblast (PROTO), (**C**) myofibroblasts (MYO) cells. Controls are represented by BJ-5ta, proto-myofibroblast or myofibroblast cells incubated with their own conditioned medium. Cells were grown for 48 h with conditioned media or SCM, and cell viability was evaluated by an ATP assay. Data are means of at least three independent experiments ± S.E. * *p* < 0.05.

We evaluated whether the potential paracrine signals, from the stromal microenvironment of our experimental system could influence the metabolic activity and cell viability of fibroblast cell types grown as monolayers. To this aim, BJ-5ta, proto-myofibroblast and myofibroblast cells were incubated with the conditioned media of all fibroblastic cell types, as well as spheroids from myofibroblasts and the standard culture medium (Figure 7).

Additionally, we incubated fibroblast cells with a standard culture medium in order to evaluate whether different quantities of serum contained in the conditioned media and standard culture medium could affect fibroblast viability.

BJ-5ta cells, proto-myofibroblasts and myofibroblasts (Figure 7) treated with the different conditioned media did not show any difference in the metabolic activity and cell viability, compared with the control.

In the light of these results, it is possible to assume that the cytotoxic activity of proto-myofibroblasts may be specific for melanoma cells, because the conditioned medium of proto-myofibroblasts did not affect the viability of BJ-5ta fibroblasts that represent the ubiquitous non-activated fibroblasts present in all body tissues.

Moreover, it is noteworthy that BJ-5ta, proto-myofibroblast and myofibroblast cells treated with a standard culture medium did not display any difference in cell viability compared with cells incubated with their own conditioned medium. In particular, this experimental evidence has indicated that diverse amounts of serum contained in conditioned media and culture standard media influence fibroblast cell viability to the same extent.

### 3.5. Comparative Analysis of Cytokines and Growth Factors levels in Proto-Myofibroblast- and Melanoma Cell-Conditioned Media

**Figure 8:**
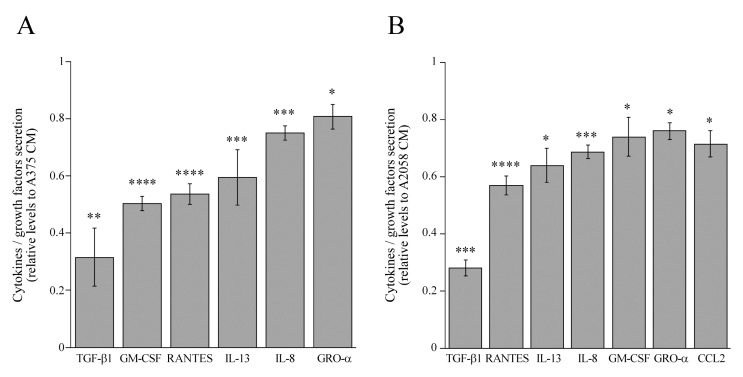
The levels of cytokines, chemokines and growth factors secreted by proto-myofibroblasts compared with the conditioned media of A375 and A2058 melanoma cells. The conditioned media of melanoma cells and proto-myofibroblasts were subjected to cytokine profiling, and a densitometric analysis of the cytokines and growth factors of the proto-myofibroblast-conditioned medium was performed. Control values, set as 1, are represented by cytokines/growth factors levels of A375 (**A**) and A2058 (**B**) melanoma cell-conditioned media. Values are represented by mean ± SE (n = 3) compared with the signal intensity of A375 (A375 CM)- and A2058 (A2058 CM)-conditioned media, set as 1. The asterisks show a statistically significant difference between melanoma cells and proto-myofibroblast-conditioned media, according Student’s *t* test (* *p* < 0.05, ** *p* < 0.01, *** *p* < 0.005, **** *p* < 0.0001).

It is known that the interaction between stromal fibroblasts and melanoma cells is regulated by cytokines and growth factors [16]. Therefore, we performed an array analysis to evaluate the cytokine and growth factor profiling of melanoma cells and proto-myofibroblast-conditioned media, and we compared the cytokine/growth factor levels of a proto-myofibroblast-conditioned medium with those of an A375 and A2058-cell conditioned media (Figure 8).

Densitometric quantification showed a decrease of some cytokines and growth factors secretion in proto-myofibroblast-conditioned medium compared with the conditioned media of melanoma cells. The conditioned medium of proto-myofibroblasts was characterized by a significantly lower secretion of TGF-β1, granulocyte–macrophage colony-stimulating factor (GM-CSF), regulated upon activation, normal T-cell expressed, and secreted (RANTES), interleukin (IL)-13, IL-8 and growth-regulated oncogene α (GRO-α) compared with the conditioned media of both A375 (Figure 8A) and A2058 cells (Figure 8B). Particularly, in the proto-myofibroblast-conditioned medium, the secretory levels of TGF-β1, GM-CSF, RANTES, IL-13, IL-8 and GRO-α showed 0.31, 0.50, 0.54, 0.59, 0.75 and 0.81 fold changes, respectively, compared with the levels of the same cytokines and growth factors secreted in the conditioned medium of the A375 cells (Figure 8A). Furthermore, the conditioned medium of proto-myofibroblasts displayed 0.28, 0.57, 0.64, 0.69, 0.74 and 0.76 fold changes of TGF-β1, RANTES, IL-13, IL-8, GM-CSF, GRO-α with respect to the A2058-conditioned medium, respectively (Figure 8B). Additionally, the conditioned medium of proto-myofibroblasts showed a 0.72-fold change of C-C motif chemokine ligand 2 (CCL2) levels with respect to the conditioned medium of A2058 cells. These results indicate that conditioned medium of proto-myofibroblasts could represent an unfavorable microenvironment hampering cell viability and migration of melanoma cells.

## 4. Discussion

Melanoma tissue is a heterogeneous network modulated by interactions between melanoma cells and the tumor microenvironment [1]. The stromal microenvironment is constituted by an ECM, fibroblasts, adipocytes, pericytes, endothelial, immune and inflammatory cells [45]. Therefore, melanoma development is associated with stromal cell recruitment and the activation of cell types, which differentiate into macrophages, mast cells, adipocytes and CAFs [46]. In particular, the activation of resident fibroblasts and their differentiation into CAFs, induced by melanoma paracrine interactions, creates a microenvironment suitable for melanoma growth, dissemination and the development of drug resistance [16]. The crosstalk between CAFs and melanoma cells furnishes the building blocks for the macromolecules biosynthesis and ATP production required for the rapid proliferation and growth of cancer cells and for sustaining the progression of metastatic melanoma [20,21]. In this scenario, the capability of cancer cells to adapt to the changes of the microenvironment and integrate stromal signals with proliferative and invasive behaviors affects the outcome of metastatic melanoma [45].

In this work, we developed an in vitro model that mimicked part of the stromal microenvironment formed by the fibroblasts, proto-myofibroblasts, myofibroblasts, and aggregates of inactivated myofibroblasts, such as spheroids. We generated proto-myofibroblasts from primary myofibroblasts forced to grow for 216 h as spheroids and then transferred to cell culture dishes to allow for the adhesion growth. In particular, the proto-myofibroblasts phenotype is characterized by a dramatic decrease of α-SMA and COX-2 levels, as well as an increase of migratory capability and cell viability, compared with myofibroblasts. Furthermore, proto-myofibroblasts, which represent the intermediate cell type during the activation process of fibroblasts into myofibroblasts [7], display a different pattern of stress fibers with respect to myofibroblasts. It is noteworthy that in our experimental system, proto-myofibroblasts showed a greater cell viability compared with both BJ-5ta and myofibroblast cells. These data suggest that myofibroblasts, during 3D culture, undergo a cell reprogramming that leads to their dedifferentiation into proto-myofibroblasts. However, we need further studies to elucidate the mechanisms that regulate the dedifferentiation of myofibroblasts into proto-myofibroblasts.

Proto-myofibroblasts localize in specialized normal connective tissue, such as alveolar septum, and in early granulation tissue of open wound [7]. The presence of stable proto-myofibroblasts in normal alveolar septa is associated with the repair process, leading to the neoformation of myofibroblasts following lung injury [47]. Proto-myofibroblasts are characterized by a proliferative and migratory program; instead, the myofibroblasts are cells that in vivo are devoted to contraction and ECM synthesis [7,10]. Furthermore, myofibroblasts in vitro can originate from fibroblasts if they are cultured on high stiffness substrate, such as plastic dishes, and they express COX-2 in the presence of a culture medium supplemented with fetal bovine serum [26].

To the best of our knowledge, this work represents the first study that combines the development of an in vitro stromal microenvironment containing proto-myofibroblasts and the study of their crosstalk with melanoma cells. The analysis of this interaction showed that the conditioned medium of proto-myofibroblasts induced cell death in both A375 and A2058 cells, even if the cytotoxic effect was more evident in A2058 cells. In our experimental system, the cytotoxic effect of the proto-myofibroblast-conditioned medium is specific for melanoma cells because it slightly alters only the cell viability and metabolic activity of myofibroblasts, but it does not modify the cell viability of BJ-5ta-inactivated fibroblasts that represent ubiquitous mesenchymal cells localized in almost all adult tissues. These data could be useful in a possible therapeutic perspective, because the unspecific cell effects of proto-myofibroblasts could have more serious consequences in ubiquitous cell types, such as fibroblasts, than in other specialized cell types [32]. Additionally, the conditioned medium of proto-myofibroblasts inhibits the migratory capability of both melanoma cell lines. However, it is noteworthy that anti-proliferative and anti-migratory signals are also elicited by the conditioned medium of both myofibroblasts and spheroids, but the conditioned medium of proto-myofibroblasts triggers a stronger anti-proliferative and anti-migratory response. On the other hand, the conditioned medium of BJ-5ta cells affects only the migration of A2058 cells. It is important to note that the addition of fresh serum in conditioned media of melanoma cells and proto-myofibroblasts did not affect the paracrine interactions of these two cell types. These results support other works that have shown the important role of fibroblasts on melanoma growth and progression also before their differentiation into CAFs. Zhou et al. reported that in a murine experimental model, normal dermal fibroblasts induced a G1/S cell cycle arrest and block mesenchymal transition of melanoma cells [15]. Conversely, dermal fibroblasts, lacking β-catenin, sustain the development of melanoma in vivo [15]. The pro-tumorigenic activity of fibroblasts is supported by the study of Xiangnan Guan et al. [17]. In particular, the prolonged CDK4/6 inhibitor treatment induces senescence and a robust senescence-associated secretory phenotype (SASP) in mouse embryonic fibroblasts [17]. The senescent fibroblasts trigger genotype-dependent proliferative responses in melanoma co-cultures, suppress the anti-tumor immune response and sustain melanoma growth in an in vivo immunocompetent murine model [17]. Moreover, it has been reported that normal human fibroblasts can influence melanoma therapeutic outcomes. In particular, co-cultures of melanoma cells with fibroblasts lead to the protection of cancer cells from BRAF inhibitor (BRAFi) such as vemurafenib [48]. The resistance is not mediated by soluble factors but is linked to tumor–stromal cell proximity. In fact, in co-cultures where the cell proximity has been disabled by a semipermeable membrane but cell communication can be mediated by soluble factors, fibroblasts fail to induce BRAFi resistance [48]. In our experimental model, the specific cytotoxic effect and the anti-migratory influence of the proto-myofibroblast-conditioned medium could be due to soluble factors or extracellular vesicles that regulate the structure and signals of the tumor microenvironment [44,49,50,51]. In particular, the array analysis of the conditioned media indicates that proto-myofibroblasts secrete significantly lower levels of some cytokines and growth factors such as TGF-β1, GM-CSF, RANTES, IL-13, IL-8 and GRO-α compared with the conditioned media of both A375 and A2058 cells. Moreover, the conditioned medium of proto-myofibroblasts also showed a significant decrease of the CCL2 level with respect to the conditioned medium of A2058 cells. These data suggest that the decrease of some cytokine and growth factor secretion could be associated with the capability of proto-myofibroblasts to hamper the cell viability and migratory capability of melanoma cells.

It is known that TGF-β regulates paracrine interaction between cancer cells and fibroblasts, and it has a relevant role during melanoma development [52]. In particular, TGF-β signaling sustains melanoma aggressiveness and metastasis [52,53]. Moreover, the TGF-β pathway is linked to tumor immune escape and affects the efficacy of anti-CTLA-4 immunotherapy [52,54]. The granulocyte–macrophage colony-stimulating factor (GM-CSF) can promote anti-tumor responses, but some preclinical data indicate that GM-CSF may sometimes promote tumor growth [55]. Moreover, in murine experimental models, GM-CSF promotes bone-marrow-derived cells mediated melanoma cell proliferation in vitro [56].

Regulated upon activation, normal T-cell expressed, and secreted (RANTES) and its receptor C-C chemokine receptor type 5 (CCR5) regulate in solid tumors the paracrine interaction between cancer cells and fibroblasts [57]. Cancer cells secrete RANTES or induce fibroblasts to secrete RANTES which interacts in a paracrine or autocrine manner on CCR5-positive tumor cells to sustain their proliferation, to recruit immunosuppressive cells (T-reg cells, monocyte), to trigger osteoclasts activation and bone metastasis, to sustain neoangiogenesis, and to support the dissemination of tumor cells. Particularly, in melanoma RANTES expression is associated with higher malignancy and increased tumor formation. Furthermore, the RANTES/CCR5 axis could sustain melanoma progression by increasing levels of immunosuppressive cells [57].

In solid tumors, the pathway regulated by interleukin-13 (IL-13) and its receptor interleukin (IL)-13Rα2 sustain migration and metastasis [58].

Interleukin-8 (IL-8) is a main regulator of the growth, angiogenesis and metastasis of melanoma in preclinical animal models. Moreover, the metastatic potential of IL-8 is associated with its ability to trigger vascularization, activate MMP-2, and increase anoikis resistance [59].

Growth-regulated oncogene α (GRO-α), also called chemokine (C-X-C motif) ligand 1 (CXCL1), is overexpressed in melanoma and is involved in melanoma growth, survival, angiogenesis and metastasis [60].

C-C motif chemokine ligand 2 (CCL2) is a chemokine that regulates the growth and pro-invasive capability of metastatic melanoma cells [61]. Therefore, the decreased levels of these cytokines and growth factors could be associated with cytostatic, cytotoxic and anti-migratory effects of the proto-myofibroblast-conditioned medium. However, we are planning and performing further studies to elucidate signals involved in the in vitro anti-tumor activity of proto-myofibroblasts, and we will evaluate the involvement of exosomes in the interaction between proto-myofibroblasts and melanoma cells.

We did not detect any effect of conditioned media on the invasiveness capability of melanoma cells, but we cannot exclude the idea that in our experimental system, fibroblasts need cancer cell proximity to influence the invasion capability of melanoma cells.

On the other hand, the conditioned media of melanoma cells neither alter the cell viability of proto-myofibroblasts nor modify the outgrowth and migratory capability of proto-myofibroblasts grown as spheroids. Conversely, BJ-5ta cell viability, and BJ-5ta and myofibroblast outgrowth, and BJ-5ta and myofibroblast migratory capability are affected by the conditioned media of melanoma cells. The influence of cancer cells on CAF differentiation, metabolism and proliferation regulates the development, progression and outcome of solid tumors. In particular, the CAF phenotype, induced by interaction with cancer cells, is characterized by alterations of proliferation rate, metabolic activity and migratory capability [6]. It has been reported that CAFs and normal fibroblasts isolated from cancer specimens and 6 cm away from tumor tissue have different proliferation rates: proliferation of CAFs is decreased compared with that of normal fibroblasts [62]. In solid tumors, the decrease of CAF proliferation is linked to a metabolic reprogramming associated with a catabolic profile that sustains the massive and uncontrolled proliferation and nutrients demand of cancer cells [6]. Therefore, catabolic CAFs are enslaved by tumor to provide energy and biomass and sustain tumor growth [6,63]. At early stages of melanoma, the interaction between cancer cells and fibroblasts aggregates leads to changes of fibroblasts phenotype such as increased migration that is a typical feature of CAFs and the formation of dermal tumor niche [4]. Conversely, in our in vitro model, the experimental evidence indicates that the interactions between melanoma cells and proto-myofibroblasts, mediated by conditioned media, did not lead to proto-myofibroblast reprogramming towards a CAF like phenotype.

In the complex, proto-myofibroblasts could represent a cell type that can counteract the effects of dermal tumor niche. Hence, proto-myofibroblasts could be useful to study new therapeutic strategies targeting melanoma growth and dissemination, although further studies will be necessary to highlight and elucidate the factors and the mechanisms responsible for potential proto-myofibroblast anti-tumor activity.

## 5. Conclusions

We have developed an in vitro experimental system that can mimic the heterogeneity and the dynamicity of fibroblast population observed in vivo during the wound healing process. The role of fibroblasts, proto-myofibroblasts and myofibroblasts in the wound healing process is well known [7]. Moreover, during wound repair, fibroblasts differentiate into myofibroblasts that can form aggregates [64]. Fibroblasts, fibroblast aggregates and myofibroblasts are present also in the stromal microenvironment of solid tumors, considered wounds that never heal [18], and they regulate cancer growth, dissemination and therapeutic resistance.

Conversely, to the best of our knowledge, proto-myofibroblasts do not belong to the population of stromal cells that interact with melanoma in vivo. Therefore, we think that the analysis of crosstalk between proto-myofibroblasts and melanoma cells is the remarkable novelty of this work. In particular, the cytotoxic and the anti-migratory activity of the conditioned medium of proto-myofibroblasts, whose phenotype is not affected by interaction with melanoma cells, will lead to performance of further studies to elucidate the mechanisms that regulate proto-myofibroblast anti-tumor activity.

## Supplementary Materials

The following are available online at https://www.mdpi.com/2073-4409/8/11/1435/s1, Figure S1: Influence of serum amounts on paracrine interaction between proto-myofibroblasts and melanoma cells. A375 (**A**) and A2058 (**B**) cells were incubated for 48 h with their own conditioned medium (Control), melanoma cell conditioned medium supplemented with 10% fresh serum (melanoma CM+), conditioned medium of proto-myofibroblasts (PROTO CM) and conditioned medium of proto myofibroblasts supplemented with 10% fresh serum (PROTO CM+). Cell viability was evaluated by ATP assay. Data are means of three independent experiments ± S.E. * *p* < 0.0001. Figure S2: Influence of serum amounts on migration of melanoma cells. A375 (**A**) and A2058 (**B**) cells were incubated for 48 h with their own conditioned medium (Control), melanoma cell conditioned medium supplemented with 10% fresh serum (Melanoma CM +), conditioned medium of proto-myofibroblasts (PROTO CM) and proto-myofibroblast -conditioned medium supplemented with 10% fresh serum (PROTO CM+). The representative images, of three independent experiments, show the same fields with scratching at 0 h, and 48 h after wounding. Scale bar 200 µm. Magnification X 10. Migratory capability quantification of A375 (**C**) and A2058 (**D**) melanoma cells. Wound widths were measured at 0 h and 48 h after wounding. Data are expressed as percentage of fold-decrease of open wound area compared to control (0 h) set as 100%, and are reported as mean of three independent experiments ± S.E. * *p* <0.05, ** *p* < 0.01, *** *p* <0.005.
