# Supplementary material for: Development of a Stromal Microenvironment Experimental Model Containing Proto-Myofibroblast Like Cells and Analysis of Its Crosstalk with Melanoma Cells: A New Tool to Potentiate and Stabilize Tumor Suppressor Phenotype of Dermal Myofibroblasts
Source: Cells. 2019 Nov 14;8(11):1435. doi: 10.3390/cells8111435 (PMC6912587; doi:10.3390/cells8111435)
Supplement: Supplementary file 1 [file cells-08-01435-s001.pdf]

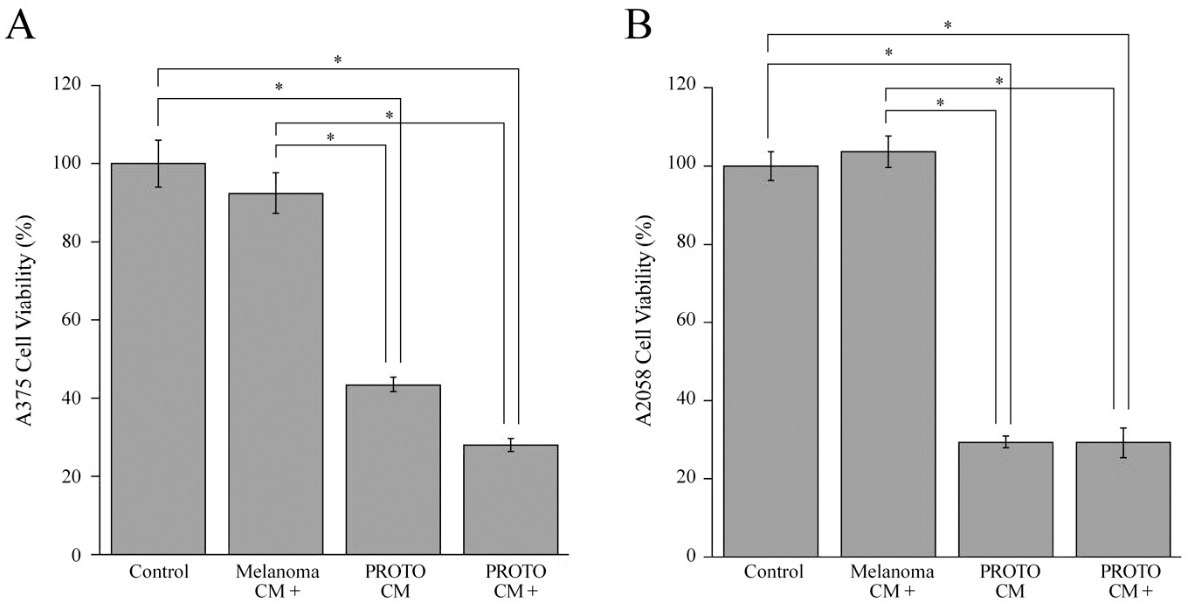

Figure S1. Influence of serum amounts on paracrine interaction between proto-myofibroblasts and melanoma cells. A375 **(A)** and A2058 **(B)** cells were incubated for 48 h with their own conditioned medium (Control), melanoma cell conditioned medium supplemented with 10% fresh serum (melanoma CM+), conditioned medium of proto-myofibroblasts (PROTO CM) and conditioned medium of proto myofibroblasts supplemented with 10% fresh serum (PROTO CM +). Cell viability was evaluated by ATP assay. Data are means of three independent experiments ± S.E . *P<0.0001.

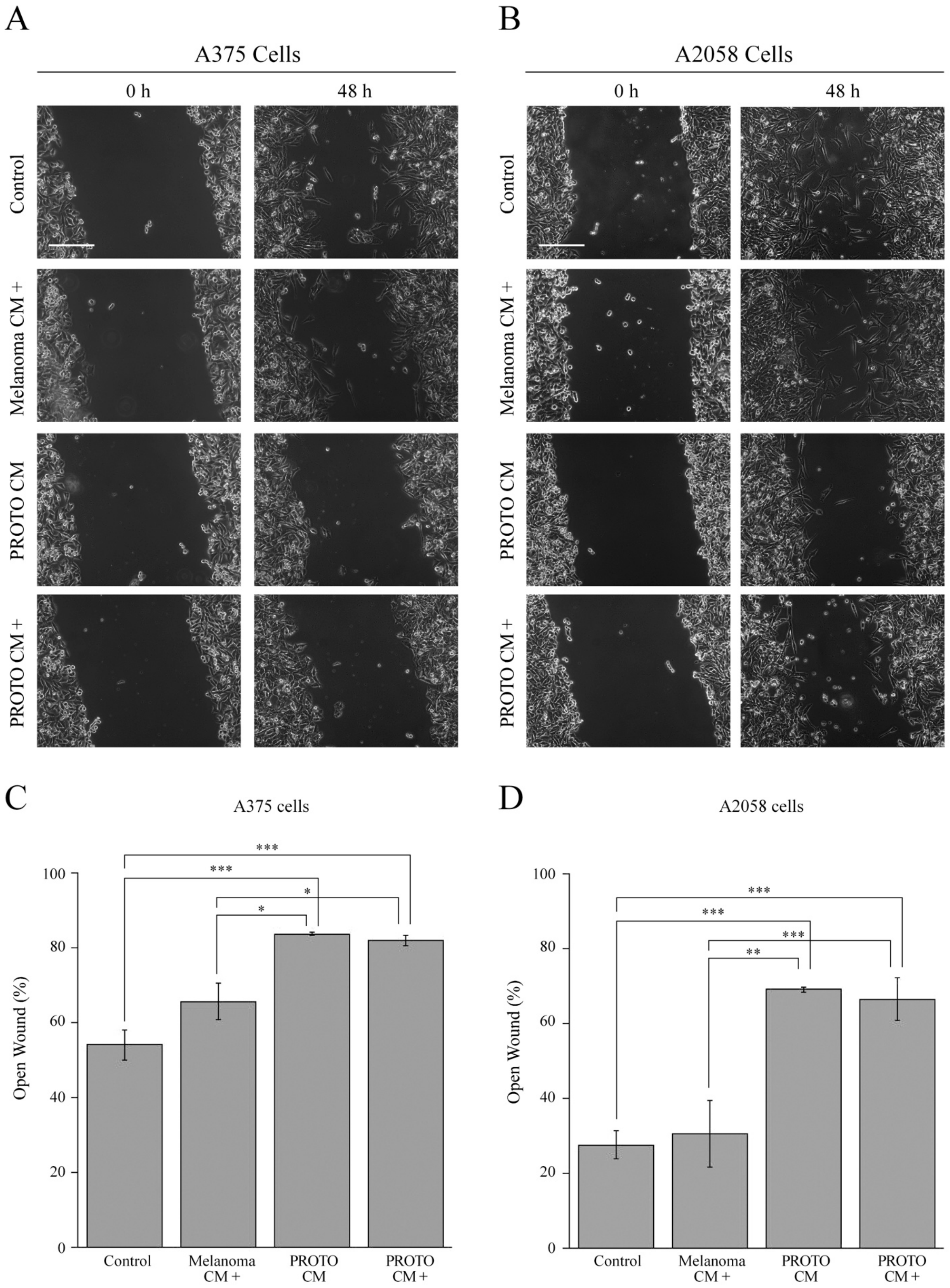

Figure S2. Influence of serum amounts on migration of melanoma cells. A375 **(A)** and A2058 **(B)** cells were incubated for 48 h with their own conditioned medium (Control), melanoma cell conditioned medium supplemented with 10% fresh serum (Melanoma CM +), conditioned medium of proto-myofibroblasts (PROTO CM) and proto-myofibroblast conditioned medium supplemented with 10% fresh serum (PROTO CM +). The representative images, of three independent experiments, show the same fields with scratching at 0 h, and 48 h after wounding. Scale bar 200 μm.

Magnification X 10. Migratory capability quantification of A375 (**C**) and A2058 (**D**) melanoma cells. Wound widths were measured at 0 h and 48 h after wounding. Data are expressed as percentage of fold-decrease of open wound area compared to control (0 h) set as 100%, and are reported as mean of three independent experiments ± S.E. *P<0.05, **P< 0.01, ***P<0.005.
